# Effect of an herbal mouthwash on periodontal indices in patients with plaque-induced gingivitis: A cross-over clinical trial

**DOI:** 10.34172/japid.2022.017

**Published:** 2022-10-08

**Authors:** Mohamadreza Talebi Ardakani, Atiyeh Farahi, Faraz Mojab, Anahita Moscowchi, Zahra Gharazi

**Affiliations:** ^1^Department of Periodontics, School of Dentistry, Shahid Beheshti University of Medical Sciences, Tehran, Iran; ^2^Department of Restorative Dentistry, School of Dentistry, Islamic Azad University of Medical Sciences, Tehran, Iran; ^3^School of Pharmacy, Shahid Beheshti University of Medical Sciences, Tehran, Iran; ^4^Traditional Medicine Researcher

**Keywords:** Gingivitis, herbal medicine, periodontal index

## Abstract

**Background.** Recent advances in alternative medicine have led to the introduction of various new herbal products for treating gingivitis as the most prevalent gingival disease. The present study clinically evaluated the effect of a herbal mouthwash consisting of 5 herbal extracts (Myrtus communis, Quercus brantii, Punica granatum, Portulaca olerace, and Boswellia serrata) on periodontal indices.

**Methods.** Fifty patients with plaque-induced gingivitis were included in this randomized, dou­ble-blinded clinical trial and divided into two groups. Following scaling and root planing (SRP), they were prescribed 0.2% chlorhexidine (CHX) (group 1) and herbal mouthwash (group 2) twice a day for 14 days. Both groups received saline mouthwash for the subsequent 14 days (wash-out time). Then, they used the mouthwashes in a cross-over manner for an additional two weeks. Probing pocket depth (PPD), gingival index (GI), bleeding on probing (BOP), and plaque index (PI) were recorded at baseline and after each period of mouthwash use. The data were analyzed by SPSS software, using generalized estimating equation (GEE) linear regression and paired t-test. P<0.05 was considered sta­tistically significant.

**Results.** Both groups exhibited statistically significant improvements in the periodontal indices compared to the baseline (P<0.05); however, there were no significant differences between the two study groups in this regard.

**Conclusion.** The experimental herbal mouthwash improved the periodontal condition in plaque-induced gingivitis after two weeks, comparable to the effect of %0.2 CHX mouthwash in terms of PPD, BOP, PI, and GI.

## Introduction

 Gingivitis is the inflammation of soft tissues around teeth without active bone loss, usually caused by the chronic presence of dental plaque.^1^ The prevalence of gingivitis varies in different countries and ages and is reported to affect approximately 80% of adults.^2-4^ The main etiology of this disease is a microbial plaque containing gram-positive aerobic/facultative anaerobic *Coccobacilli*.^5,6^

 Treatment of periodontal diseases is routinely based on non-surgical mechanical debridement, including SRP by hand instruments and/or ultrasonic devices, and appropriate maintenance of oral hygiene. The efficacy of mouthwashes in areas with limited access to plaque removal and a higher possibility of recolonizing microorganisms has previously been documented.^7,8^ In addition, bacterial accumulation in other areas of the oral cavity, such as the tongue, oral mucosa, and tonsils, can contribute to re-infection.^9,10^Antimicrobial mouthwashes may reduce bacterial adhesion to the tooth surfaces or the growth and division of microorganisms.^11^

 Chlorhexidine (CHX) is currently the best-known antimicrobial agent to prevent and treat gingival diseases.^12^ However, its long-term use can lead to side effects such as discoloration of teeth and restorations, taste disturbances, and allergic reactions.^13^Thus, herbal alternatives are generally preferred to chemicals because they are available at lower prices and have no or fewer side effects.^11^

 Herbs have been used as medicine for centuries and have biological activities, including antioxidant and antimicrobial effects.^14,15^ A herbal mouthwash made by combining five medicinal herbs (*M yrtus communis*, *Q uercus brantii*, *Punica granatum*, *Portulaca olerace, *and *Bo swellia serrata*), traditionally used to relieve oral infections in southern Iran for centuries. Considering the available evidence on anti-inflammatory, antibacterial, antioxidant, antifungal, and antiviral effects of the herbal mouthwash constituents.^7,9,14,16-21^ this study aimed to assess the effect of this mouthwash as an adjunct to the non-surgical treatment of plaque-induced gingivitis.

## Methods

 This randomized, double-blind controlled clinical trial was performed at the Department of Periodontics, School of Dentistry, Shahid Beheshti University of Medical Sciences, Tehran, Iran, in 2018. This study was conducted following the 1964 Helsinki declaration, following approval by the Ethics Committee of Shahid Beheshti University of Medical Sciences (IR.SBMU.DRC.REC.1398.217) and the Iranian Registry of Clinical Trials (IRCT20190712044177N2). Written informed consent was obtained from all patients before their enrollment.

 Our samples were selected randomly from patients diagnosed via clinical and radiographic evaluation with generalized plaque-induced gingivitis involving at least three quadrants with 20 teeth. Patients were excluded if they had any of the following criteria: (I) pregnancy or lactation, (II) systemic diseases, (III) taking any drug affecting the periodontium, including immunosuppressants or those causing gingival overgrowth, (IV) antibiotic therapy during the last six months, (V) smoking, (VI) periodontal treatment during the last three months, (VII) severe caries or extensive restoration on the objective teeth, (VIII) orthodontic treatment during the study, (IX) history of allergy to the tested materials, (X) non-cooperative patients or poor oral hygiene. The sample size was estimated at 25 participants in each group based on the study of Yaghini et al, SD: 0.46, 0.63, and at least one unit of expected difference between the two groups.^9^ Fifty volunteers 20‒30 years of age were selected from dental students in Shahid Beheshti School of Dentistry by a single trained periodontist (M. TA.).

 The herbal mouthwash was prepared by a pharmacist (F.M.) by combining equal amounts (100 grams) of each fresh plant (*M yrtus communis L., Q uercus brantii Lindl., Punica granatum L., Portulaca oleracea L.,*and* Bo swellia serrata Roxb*) according to the traditional recipe. The plants were powdered and macerated with 96% ethanol (X3) separately; the ethanol was evaporated, and the mouthwash was prepared by mixing the extracts (400 mg each and a total of 2%) in water and 3% ethanol. The extraction procedure was then continued to attain a clear herbal-smelling monophasic solution containing 3% alcohol as the solvent.

 All the patients underwent SRP using an ultrasonic device (Mectron-Carasco GE, Italy) and hand instruments (Hufriedy, Chicago, Illinois, USA) and were instructed to brush using the modified Bass technique. They were advised not to use any other mouth rinses during the experiment. The subjects were randomized into two cohorts (A: CHX/herbal and B: herbal/CHX) by generating a random number in Microsoft Excel Software. Group A was given 0.2% CHX (Iran Najo Pharmaceutical Co., Tehran, Iran), and group B was given a herbal mouthwash. The solutions were presented in dark bottles to ensure that patients and researchers were blinded to the type of mouthwash. The bottles were distributed among the individuals by a clinical examiner (A.F.) blinded to the contents of the containers. All clinical examinations were conducted by a blinded examiner (A.F.). The patients rinsed their mouths with a fixed amount of the solutions twice daily for 90 seconds after brushing for two weeks. Clinical indices including GI,^22^ PI,^23^ BOP,^24^ and PPD in the buccal, mesial, distal, and lingual aspects were recorded for all teeth at baseline and after two weeks. All the measurements were performed by a single masked examiner using a Michigan probe (Hufriedy, Chicago, Illinois, USA). The wash-out duration was 14 days, using a saline solution as the mouthwash. Then, groups A and B were given the herbal and 0.2% CHX mouthwashes, respectively, in a cross-over manner; the indices were measured at the beginning of mouthwash use and two weeks later.

 At each examination, the patients were asked about the side effects of herbal mouthwash, such as discoloration of teeth and restorations, taste disturbance, and allergic reactions.

###  Statistical analysis

 Data were analyzed using SPSS 17 software (SPSS Inc., Chicago, IL, USA). GEE linear regression was conducted to evaluate the effect of mouthwash on the indicators by controlling the effect of the initial amount before the start of treatment and arranging mouthwashes. Paired t-test was used to compare changes in periodontal indices by two types of mouthwash. P<0.05 was considered statistically significant.

###  Result

 The present randomized clinical trial was performed on 25 dental students (13 females and 12 males), with a maximum age of 24 years (44%). As presented in Tables 1 and 2, all the evaluated parameters decreased after using 0.2% CHX and herbal mouthwashes in all cases.

**Table 1:** Clinical indices at baseline and two weeks after using 0.2% CHX mouthwash

	**PI**	**GI**	**BOP**	**PPD (M)**	**PPD (L)**	**PPD (D)**	**PPD (B)**
**Baseline** **Mean(SD)**	0.602 (0.2669)	0.912 (0.4386)	0.357 (0.2057)	1.906 (0.400)	1.369 (0.3982)	2.018 (0.4236)	1.224 (0.2289)
**After 2 weeks** **Mean(SD)**	0.433 (0.2707)	0.424 (0.2962)	0.152 (0.1438)	1.578 (0.4067)	1.300 (0.3159)	1.658 (0.4263)	1.104 (0.1291)

PI: plaque index, GI: gingival index, BOP: bleeding on probing, PPD: probing pocket depth, M: mesial, L: lingual, D: distal, B: buccal, SD: standard deviation

**Table 2:** Clinical indices at baseline and two weeks after using the herbal mouthwash

	**PI**	**GI**	**BOP**	**PPD (M)**	**PPD (L)**	**PPD (D)**	**PPD (B)**
**Baseline** **Mean(SD)**	0.585 (0.2257)	0.803 (0.4745)	0.339 (0.2588)	1.789 (0.4243)	1.311 (0.3358)	1.843 (0.4070)	1.237 (0.2264)
**After 2 weeks** **Mean(SD)**	0.396 (0.1975)	0.381 (0.3459)	0.116 (0.1287)	1.456 (0.3102)	1.213 (0.3259)	1.510 (0.3497)	1.108 (0.1788)

PI: plaque index, GI: gingival index, BOP: bleeding on probing, PPD: probing pocket depth, M: mesial, L: lingual, D: distal, B: buccal, SD: standard deviation

 No significant difference was observed between the two types of mouthwash between the baseline and follow-up evaluations: GI (P=0.809), BOP (P=0.292), PI (P=0.595), PPD mesial (P=0.177), PPD distal (P=0.114), PPD lingual (P=0.477), PPD buccal (P=0.966) (Table 3). There was also no significant difference in terms of the two modes of herbal and 0.2% CHX mouthwash use (P>0.05).

**Table 3:** Comparison of the changes between chlorhexidine and herbal mouthwashes

	**Mean**	**Std.Deviation**	**P-value**
**BOP**	-0.0188	0.26080	0.7220
**GI**	0.06660	0.62160	0.5970
**PPD (buccal)**	0.0090010	0.2170210	0.8370
**PPD(mesial)**	0.00510	0.52450	0.9620
**PPD(distal)**	-0.0271	0.55430	0.8090
**PPD(lingual)**	0.02850	0.18730	0.4550
**PI**	0.01960	0.28100	0.7300

PI: plaque index, GI: gingival index, BOP: bleeding on probing, PPD: probing pocket depth

## Discussion

 Since the main etiologic factors related to the onset of gingival and periodontal diseases are facultative/strictly anaerobic *Coccobacillus* bacteria, the targeted treatment modalities aim to reduce these bacterial agents. Chlorhexidine is an antibacterial agent known as the gold standard in the prevention and treatment of gingival inflammation adjunctive to routine mechanical instrumentation.^21,25^ However, various plant products, including different vegetable oils or some aromatic molecules, have been reported to have antibacterial and protective effects on the oral environment.^26^ A herbal mouthwash containing five medicinal plants has been used for a long time in southern Iran as a remedy for chronic gingival bleeding. The present study aimed to evaluate the effectiveness of this mouthwash compared to 0.2% CHX.

 The results showed that periodontal parameters improved following the use of the herbal mouthwash, but its effect was less than that of 0.2% CHX except for the two parameters, GI and PPD (D). However, this difference between the effect of CHX and herbal mouthwash was not statistically significant, which may be attributed to the synergistic and significant antibacterial activities of the herbal extracts in the experimental mouthwash.

*Portulaca oleracea* has long been known as an antipyretic, antiseptic, antibacterial, antioxidant, and anti-inflammatory material effective in wound healing. This plant contains terpenoids, fatty acids, alkaloids, flavonoids, and minerals. Bhat et al,^14^ in a study on five medicinal plants, including *Portulaca oleracea*, concluded that it has a deterrent effect on *Pseudomonas aeruginosa*, *Bacillus subtilis*, and *Staphylococcus*.

 In 2003, Satravaha et al^27^ evaluated the effect of *Centella asiatica* and *Punica granatum *extracts as periodontal adjuvant therapies. They used these extracts as chips in deep periodontal pockets, and the results showed a significant improvement in the pocket depth and attachment level compared to the control group. It has also been shown that Punica granatum extract reduces the amount of bacterial plaque and pocket depth after three months, attributed to a significant decrease in IL-1β and IL-6 levels.^28^Mehta et al^12^ reported that *Punica granatum* inhibits oral pathogenic bacteria such as *Streptococcus mitis* and *Streptococcus mutans *but does not affect *Porphyromonas gingivalis* and *Prevotella intermedia*. The antimicrobial effect of the essential oil extract of the *M yrtus communis* plant on *P. gingivalis* has been reported.^7^ Houshmand et al^16^ investigated the effect of the *Myrtus communis* extract on some bacteria in the oral cavity. The results showed that the extract of this plant in different concentrations had different antibacterial effects on the microbial flora of the mouth; the most effective concentration against *Pseudomonas aeruginosa* was 2.5%.

 Studies have also shown that *Boswellia serrate* has a significant, negative effect on the growth of *Streptococcus mutans*, *P. gingivalis*, and *Fusobacterium nucleatum*.^19^ Raja et al^19^ found that acetyl-11-keto-b-boswellic acid (AKBA) in the extract of this plant showed significant antibacterial activity against oral pathogens.

 An in vitro study evaluated the effect of an aqueous-alcoholic mouthwash of herbal origin on *Aggregatibacter actinomycetemcomitans* (*A.a*). The results showed that despite the strong effect of this compound on *A.a*, it was less effective than 0.2% CHX as a standard mouthwash used in periodontal problems.^20^

 In general, the results of this study were consistent with the previous in vitrostudies proving the strong antimicrobial effects of herbal mouthwash constituents.^30^ The herbal mouthwash with its effective constituents against a variety of periodontopathogens seems to be a valuable adjunctive treatment modality in chronic or recurrent forms of gingivitis, especially in cases where the host immune defense is not capable of the resolution of inflammation due to a systemic disorder or a local factor which impairs the access for plaque control.^31^ All the samples selected in this study were among dental students with an acceptable baseline plaque control; however, they suffered from generalized persistent gingivitis despite a meticulous professional SRP. As no patients reported any adverse effects due to the use of the mouthwash, in addition to the comparable improvement in periodontal indices, it seems that the herbal mouthwash would be a safe and promising substitute for chlorhexidine.

###  Suggestions and limitations

 This study has limitations, such as the problems of follow-ups and patient cooperation. It is recommended that this study should be performed as a prospective randomized clinical trial with negative control and long-term follow-ups with a larger sample size to assess the long-term side effects of the herbal mouthwash. The impact of the herbal mouthwash should also be evaluated on other forms of oral infections. The impact of different aqueous or acetone solvents while preparing the plant extracts must also be tested in future studies.

## Conclusion

 The experimental herbal mouthwash improved the periodontal condition in plaque-induced gingivitis after two weeks, comparable to the effect of 0.2% CHX mouthwash in terms of PPD, BOP, PI, and GI.

## Acknowledgments

 None.

## Competing interests

 The authors declare no competing interests related to the publication of this work.

## Authors’ contributions

 Design of the work: MTA.; Methodology: FM; Acquisition of data: AF; Drafting the work: AF; Revision: AM. All authors read and approved the final manuscript.

## Funding

 The work was supported by a grant from the Research Council of Shahid Beheshti University of Medical Sciences, Tehran, Iran.

## Availability of data

 The data that support the findings of this study are available from the corresponding author upon reasonable request.

## Ethics approval

 This study was approved by the Ethics Committee of Shahid Beheshti University of Medical Sciences (IR.SBMU.DRC.REC.1398.217) and the Iranian Registry of Clinical Trials (IRCT20190712044177N2.
